# Subtle Role for Adenylate Kinase 1 in Maintaining Normal Basal Contractile Function and Metabolism in the Murine Heart

**DOI:** 10.3389/fphys.2021.623969

**Published:** 2021-03-31

**Authors:** Sevasti Zervou, Debra J. McAndrew, Hannah J. Whittington, Hannah A. Lake, Kyung Chan Park, Kuan Minn Cha, Philip J. Ostrowski, Thomas R. Eykyn, Jürgen E. Schneider, Stefan Neubauer, Craig A. Lygate

**Affiliations:** ^1^Division of Cardiovascular Medicine, Radcliffe Department of Medicine, Wellcome Centre for Human Genetics, University of Oxford, Oxford, United Kingdom; ^2^British Heart Foundation Centre for Research Excellence, University of Oxford, Oxford, United Kingdom; ^3^Department of Physiology Anatomy and Genetics, University of Oxford, Oxford, United Kingdom; ^4^British Heart Foundation Centre for Research Excellence, King’s College London, St. Thomas Hospital, London, United Kingdom; ^5^Experimental and Preclinical Imaging Centre (ePIC), Leeds Institute of Cardiovascular and Metabolic Medicine, University of Leeds, Leeds, United Kingdom

**Keywords:** adenylate kinase, energy metabolism, creatine kinase, ischaemia/reperfusion injury, cardiac energetics

## Abstract

**Aims:**

Adenylate kinase 1 (AK1) catalyses the reaction 2ADP ↔ ATP + AMP, extracting extra energy under metabolic stress and promoting energetic homeostasis. We hypothesised that increased AK1 activity would have negligible effects at rest, but protect against ischaemia/reperfusion (I/R) injury.

**Methods and Results:**

Cardiac-specific AK1 overexpressing mice (AK1-OE) had 31% higher AK1 activity (*P* = 0.009), with unchanged total creatine kinase and citrate synthase activities. Male AK1-OE exhibited mild *in vivo* dysfunction at baseline with lower LV pressure, impaired relaxation, and contractile reserve. LV weight was 19% higher in AK1-OE males due to higher tissue water content in the absence of hypertrophy or fibrosis. AK1-OE hearts had significantly raised creatine, unaltered total adenine nucleotides, and 20% higher AMP levels (*P* = 0.05), but AMP-activated protein kinase was not activated (*P* = 0.85). ^1^H-NMR revealed significant differences in LV metabolite levels compared to wild-type, with aspartate, tyrosine, sphingomyelin, cholesterol all elevated, whereas taurine and triglycerides were significantly lower. *Ex vivo* global no-flow I/R, caused four-of-seven AK1-OE hearts to develop terminal arrhythmia (cf. zero WT), yet surviving AK1-OE hearts had improved functional recovery. However, AK1-OE did not influence infarct size *in vivo* and arrhythmias were only observed *ex vivo*, probably as an artefact of adenine nucleotide loss during cannulation.

**Conclusion:**

Modest elevation of AK1 may improve functional recovery following I/R, but has unexpected impact on LV weight, function and metabolite levels under basal resting conditions, suggesting a more nuanced role for AK1 underpinning myocardial energy homeostasis and not just as a response to stress.

## Introduction

Adenylate kinase (AK) is a key enzyme in energy homeostasis involved in the synthesis, equilibration and regulation of adenine nucleotides (Dzeja et al., 2007b). In the heart, the cytosolic isoform AK1 accounts for 90% of total AK activity (Pucar et al., 2002; Dzeja and Terzic, 2009), with localised contributions from AK2 and AK3 (mitochondria membrane and matrix), AK6 (nucleus), ecto-AK (extracellular space), and AK1β (plasma membrane) (Lee et al., 1998; Pucar et al., 2000).

AK1 catalyses the reversible reaction 2ADP ↔ ATP + AMP (Dzeja et al., 1998, 1999) thereby allowing interchange of a phosphoryl group between adenine nucleotides (i.e., AMP, ADP, and ATP, the sum of which is termed total adenine nucleotides or TAN pool) (Dzeja et al., 1985). This complements the creatine kinase (CK) system by providing a secondary mechanism for intracellular phosphotransfer.

During metabolic stress AK1 serves two additional and unique functions. Firstly, it provides a mechanism to double the energy available from ATP by utilising the β-phosphoryl group to wring one additional ATP out of two ADP molecules (Dzeja et al., 1985). Secondly, the AMP generated acts as a metabolic signal to activate adenosine monophosphate-activated protein kinase (AMPK), which is a master regulator, promoting energy production and reducing energy consumption via multiple signalling cascades (Frederich and Balschi, 2002; Dzeja et al., 2007b). In this way, AK can be said to monitor cellular metabolic state, generating signal via AMP when energy supply is limited, thereby promoting a return to energy homeostasis (Dzeja and Terzic, 2009).

The role of AK under stress conditions has been investigated previously. The AK contribution to total phosphotransfer in the healthy heart is ∼10% (compared to 89% for creatine kinase) (Dzeja et al., 1999) however, in the dog model of pacing-induced heart failure, phospho-transfer via AK increased by 134%, contributing 21% of the total ATP turnover, while phosphotransfer via CK was reduced by 52% (Dzeja et al., 1999). Similar observations have been made in murine models of heart failure due to myocardial infarction and pressure-overload, whereby CK activity was reduced while AK activity remained unchanged, suggesting that the relative importance of AK increases under stress conditions (Aksentijevic et al., 2010).

Mice deficient in cardiac AK1 have broadly normal levels of adenine nucleotides, γ-ATP turnover and CK phosphotransfer. However, under hypoxic conditions AK1 deficiency resulted in lower levels of ATP and reduced production of adenosine (Pucar et al., 2000). Further studies in *ex vivo* ischaemia/reperfusion (I/R) protocols found that hearts from AK1 deficient mice stopped beating more rapidly at onset of ischaemia and had impaired recovery of coronary flow as a consequence of reduced adenosine release (Dzeja et al., 2007a). Excess AMP would normally be degraded to adenosine by 5′-nucleotidase, which is then lost from the cell by diffusion to act as a coronary vasodilator (Ingwall, 2002).

We therefore hypothesised that increasing AK1 by genetic overexpression in the heart would be cardioprotective in I/R injury by preserving ATP levels during the ischaemic phase, by elevating intracellular [AMP] to activate AMPK, and by providing more AMP for adenosine release, all of which are known to be cardio-protective (Paiva et al., 2010). However, there is a trade-off, since increased AMP degradation to adenosine is likely to deplete the TAN pool, which might be detrimental to functional recovery. To resolve these conflicting effects, we created cardiac-specific AK1 overexpressing mice (AK1-OE), performed baseline phenotyping, and tested in models of I/R both *ex vivo* and *in vivo*.

## Materials and Methods

### Mouse Husbandry

All animal experiments were approved by the Committee for Animal Care and Ethical Review at the University of Oxford and comply with Home Office Regulations incorporating the Animals (Scientific Procedures) Act of 1986 and Directive 2010/63/EU of the European Parliament. Mice were maintained in individually ventilated cages on a 12 h night/day cycle with controlled temperature (21°C) and humidity. Mice were fed irradiated Global Diet 2916 (Envigo Research model services, United Kingdom) and water *ad libitum* and housed with littermates in specific pathogen-free conditions.

### Generation and Breeding of AK1-OE Mice

Mouse AK1 open reading frame (668 bp) with a hemagglutinin (HA) tag, was cloned into *Sal*I and *Hin*dIII cloning sites of the αMHC vector as described previously (Whittington et al., 2018), before sub-cloning of the αMHC-AK1-HA-polyA fragment into the integrase mediated cassette exchange vector, CB92 (Supplementary Figures 1A,B and Supplementary Material). This vector was then transfected into embryonic stem cells together with PhiC31 integrase and recombinant clones were obtained which harboured the transgenes within the Rosa26 locus. Recombinant clones were then injected into blastocysts and chimaeras were generated. For detailed methods please see Supplementary Material.

This strain was backcrossed for > 10 generations using C57BL/6JOlaHsd mice obtained from Envigo Research model services (United Kingdom) and are therefore congenic to C57BL/6JOlaHsd. The transgenic line was maintained through heterozygote x heterozygote breeding. For all experiments, transgenic mice were homozygote for AK1 overexpression (AK1-OE; AK1/AK1) and wild-type (WT/WT) were non-transgenic littermates bred in our establishment.

### Mouse Genotyping

DNA extraction method is included in Supplementary Material. A multiplex PCR protocol was designed to detect both the presence of AK1 transgene and zygosity status in AK1-OE mice (Supplementary Figure 1C). For detecting the AK1-specific transgene, the AK1_For and AK1_Rev pair of oligonucleotides (Supplementary Table 1) bind a sequence on the αMHC promoter, providing a PCR amplicon of 298 bp. For detecting zygosity, the Rosa26_F and Rosa26_R flanking oligonucleotides were used (Supplementary Table 1) to produce a PCR product at 508 bp. Positive pups were wild-type (WT/WT) if they produced a band at 508 bp (presence of WT Rosa26 allele), heterozygote WT/AK1 if they yielded PCR products both at 508 and 298 bp (WT Rosa26 and AK1), or homozygote AK1/AK1 if they only yielded a band at 298 bp (AK1 gene) (Supplementary Figure 1C).

### Tissue Extraction and Biochemistry

#### HPLC

Snap-frozen, powdered ventricular tissue was prepared for quantification of creatine by HPLC, and then normalised to protein content using the Lowry method as previously described (Teerlink et al., 1993). For measurement of creatine and TAN pool, a group of WT/WT mice (*n* = 16; 7M/9F) were compared to age matched AK1/AK1 (*n* = 15; 8M/7F), both at mean age of 10 weeks. Adenosine release was measured in the perfusate collected during the first 10 min of reperfusion and is expressed as nmol/mg of heart.

#### Citrate Synthase, Creatine Kinase (CK) Activity, and CK Isoenzyme Composition

Citrate synthase, creatine kinase (CK) activity, and CK isoenzyme composition were measured as previously described (Neubauer et al., 1995; Whittington et al., 2018). For both enzymatic assays, LV from *n* = 14 WT/WT and *n* = 15 age matched AK1/AK1 were harvested (WT:7M/7F; OE:7M/8F mean age 10 weeks).

#### AK1 Activity

Adenylate kinase (AK) activity was measured in LV homogenates of *n* = 6 per genotype (3M/3F; mean age 14 weeks; average age 13.7 weeks for WT/WT and 14.5 weeks for AK1/AK1) as described previously (Aksentijevic et al., 2010; details in Supplementary Material).

### Protein Expression and AMPK Phosphorylation

AK1 protein was detected in LV samples from *n* = 8 WT/WT (4M/4F; 13.5 weeks), *n* = 7 WT/AK1 (4M/3F; 12.4weeks), and *n* = 4 AK1/AK1 (4M; 8.6 weeks), as described before (Whittington et al., 2018). AK2 and AK3 protein were also detected by immunoblotting *n* = 5 each WT (2M/3F) and AK1/AK1 (OE; 3M/2F). For determining AMPK phosphorylation levels, specific cohorts of WT/WT (*n* = 13 5M/8F average age 14.5 weeks) and AK1/AK1 *n* = 15 (5M/10F age matched; mean 14.5 weeks) mice were used (detailed methods in Supplementary Material).

### ^1^H NMR Metabolomics

Heart was removed under deep isoflurane anaesthesia (4% in oxygen throughout), briefly washed in saline, blotted on tissue, and the atria and vessels removed. Remaining LV + RV tissue was snap-frozen using Wollenberger tongs. All mice were male WT/WT and AK1/AK1 (*n* = 8 each) aged 20–27 weeks (average 25 weeks). Methods are detailed in Supplementary Material.

### Hypertrophic Gene Expression

Marker genes were studied in male, *n* = 4–8 WT/WT and *n* = 4 AK1/AK1 with age range 16–17 weeks. Males were used since an increased LV weight was observed in this sex. See Supplementary Material for methods and Supplementary Table 1 for oligonucleotides used.

### Histology for Fibrosis and Myocyte Cross-Sectional Area (CSA)

For fibrosis analysis, AK1/AK1 (*n* = 4) and WT/WT (*n* = 5) LVs (all male mice, 17–18 weeks of age) were harvested whereas for measuring myocyte CSA, LVs (*n* = 4 WT/WT and *n* = 4 AK1/AK1, all male, age range 11–18 weeks) were collected and processed as described in Supplementary Material.

### Image Analysis

For fibrosis, all datasets (75–110 images per left ventricle) were processed for optimal contrast and brightness and analysed using Media Cybernetics Image Pro 10. Images were analysed using smart segmentation applying haemocytometre calibration giving areas (μm^2^) of interstitial fibrosis in blue and all other tissue including perivascular fibrosis red allowing percentage of blue interstitial fibrosis across LV to be calculated.

For CSA, 55–157 images per sample were analysed using ImageJ (v1.50i NIH). Cardiomyocytes in clear cross-section with sharp edges were selected for analysis, carefully outlined and area measured (μm^2^). Calibration was carried out using a haemocytometre.

### Tissue Water Content Calculation

WT/WT mice were *n* = 14F, *n* = 16M at 28 ± 4 and 22 ± 2 weeks, respectively. AK1/AK1 were *n* = 16F and *n* = 16M 27 ± 4 and 22 ± 2, respectively. LV was harvested blotted and weighted, then placed in oven at 55°C and weighed daily until no further weight change, difference between start and end weights was taken as the water content.

### LV Haemodynamics

Methods were as described previously, using a 1.4F Millar micro-tip cannula in free-breathing isoflurane anaesthetised mice (1.5% in medical oxygen) (Lygate et al., 2012). Mean age for all groups was 17 weeks (range 14–19). One female WT was excluded due to the presence of severe LV hypertrophy (LV weight = 113 mg; which was > 4 SD higher than the group mean) and two female WT were excluded since they developed persistent arrhythmias throughout the protocol. This left *n* = 9 mice in the female groups and *n* = 10 mice in the male groups completing the study.

### *Ex vivo* I/R

Mice were anaesthetised with sodium pentobarbital (55 mg/kg I.P.) and heparin (300 IU), hearts were excised rapidly, cannulated and perfused in Langendorff mode with standard Krebs–Henseleit buffer as described previously (Whittington et al., 2018). LV function was assessed in spontaneously beating hearts using a water-filled intraventricular balloon connected to a pressure transducer, coupled to a Powerlab chart recorder (ADInstruments, United Kingdom). Hearts were allowed to equilibrate for 15 min, followed by 20 min global no-flow ischaemia and 30 min reperfusion. This cohort consisted of *n* = 7 per genotype (both 2M/5F; average age 27–29 weeks). A separate cohort was used to measure adenosine release during the first 10 min of reperfusion under identical conditions. This consisted of male mice *n* = 10 WT/WT and *n* = 8 AK1/AK1 with average age 36–38 weeks. Perfusate was collected continuously during reperfusion and 1 mL aliquots of total perfusate were frozen for adenosine quantification by HPLC from all but one WT sample, which was accidentally lost. After 10 min reperfusion, hearts were blotted, weighed, and snap-frozen in liquid N_2_ for determination of TAN pool and total creatine by HPLC.

### *In vivo* I/R

General anaesthesia was induced in an anaesthetic chamber using 4% isoflurane in 100% medical oxygen and buprenorphine analgesia administered subcutaneously (1 mg/Kg). Mice were then intubated via the oropharyngeal route for mechanical ventilation and maintained on 2% isoflurane in oxygen throughout (Whittington et al., 2018). A thoracotomy was performed and the left anterior descending coronary artery occluded for 45 min. Reperfusion was confirmed by visual inspection and mice allowed to recover for 24 h, at which time the heart was removed under terminal isoflurane anaesthesia (4% in oxygen) and stained using phthalocyanine blue and triphenyltetrazolium chloride as described previously (Bohl et al., 2009; Whittington et al., 2018). Hearts were analysed from *n* = 13 WT/WT (6F/7M; average age 17 weeks) and AK1/AK1 *n* = 15 (8F/7M; average age 17 weeks).

### Data Analysis

Measurements and analysis were performed blind to genotype. Comparison between two genotypes was by unpaired Student’s *t*-test using Welch’s correction where variances were found to be significantly different. Comparison between 3 genotypes for mRNA and protein expression was by one-way ANOVA with Dunnett’s *post hoc* test for multiple comparisons vs. the wild-type group. *Ex vivo* function was analysed by two-way ANOVA followed by Holm-Sidek method to compare means at each time-point (GraphPad Prism v8).

## Results

### Validation of the Transgenic Model

The novel AK1-OE mouse line, Gt(ROSA)26Sor^TM 1(*Myh*6– *AK*1)^, displayed Mendelian inheritance pattern (Chi square test 0.213; *P* = 0.64) and development was normal, as suggested by similar body weight and tibial lengths in age-matched WT and OE for both female and male animals (Supplementary Table 4).

Overexpression of αMHC-AK1-HA *in vivo* was verified by testing transcript levels for both transgenic AK1 and total AK1. Transgenic AK1 mRNA was absent from WT mice and profoundly expressed in both AK1 heterozygotes (WT/AK1) and homozygotes (AK1/AK1) (*P* = 0.0014 and *P* < 0.0001, respectively) (Supplementary Figure 2A). In addition, there was a gene dosing effect as shown by an increase in transgene expression levels between WT/AK1 and AK1/AK1 (*P* = 0.03). As a result, total AK1 mRNA was elevated in the WT/AK1 and AK1/AK1 compared to WT/WT (*P* = 0.0179 and *P* < 0.0001, respectively) (Supplementary Figure 2B). Endogenous mRNA was found to be unchanged (Supplementary Figure 2C). Tissue panel analysis of total mRNA showed increase only in LV and atria (*P* = 0.004 and 0.0007, respectively) as a result of AK1-αMHC overexpression, whereas expression levels were near the limit of detection for lung and skeletal muscle, and no differences were observed in kidney, liver, or brain AK1 transcript (Supplementary Figure 2D).

Western blot of LV homogenates from WT/WT, WT/AK1, and AK1/AK1 mice, showed a trend for higher AK1 protein levels in AK1-OE vs. WT/WT (Supplementary Figures 2E,F; *P* = 0.1) and identified as a protein band at 25 kDa. The additional higher-MW protein band in OE, was also detected, as predicted by antibody specification. This indicates a mild overexpression model near the limits of detection by Western blotting. AK2 and AK3 protein levels were also examined in LV of WT and AK1/AK1 mice (Supplementary Figures 2G,H for AK2 and Supplementary Figures 2I,J for AK3). While AK2 remained unchanged, there was a significant drop in AK3 protein levels (*P* = 0.0013) in line with AK1 overexpression in LV. However, mRNA was unchanged for both AK2 and AK3 in the AK1-OE vs. WT (Supplementary Figures 2K,L, respectively).

**FIGURE 1:**
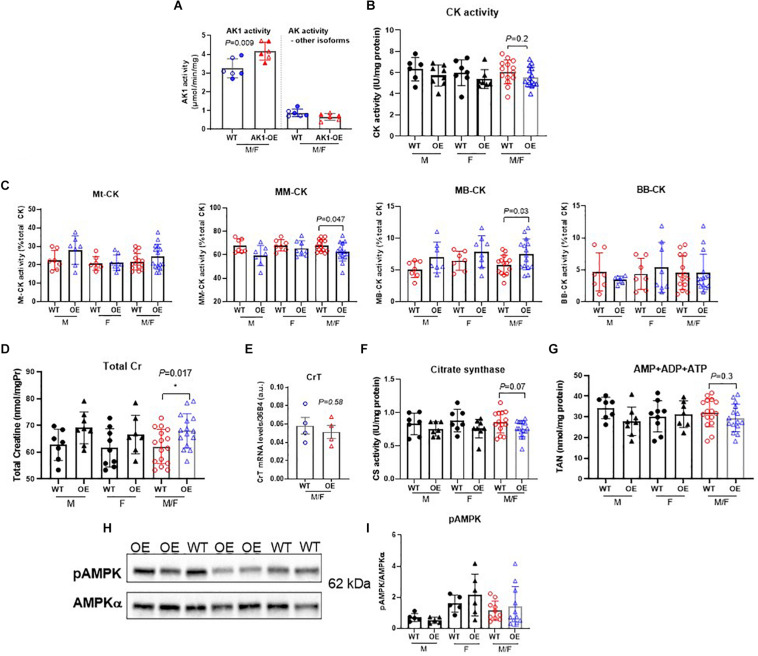
Biochemical characterisation of relevant metabolic pathways in left ventricle from WT and homozygous AK1-OE mice. AK1 protein activity was 31% higher than WT without affecting the activity of other AK isoforms; n = 6 (3M shown by solid symbols/3F) **(A)**. Total activity of creatine kinase was not altered (**B**; coloured symbols show combined M/F; WT:6M/7F, OE:8M/7F), but there was a subtle shift in isoenzyme activities from MM to MB **(C)**. For CK activities, values for M and F are shown separately, in addition to combined M/F (last 2 columns). For Mt-CK, MM-CK, MB-CK and BB-CK: WT: 7M/7F; OE: 7M/8F. Total creatine levels were slightly elevated in AK1-OE (AK1/AK1) hearts (WT 7M/9F, OE 8M/7F) (**D**; last 2 columns are combined M/F) but no changes were seen in creatine transporter (CrT) mRNA **(E)** (*n* = 4 M). Citrate synthase activity was unchanged (**F**; last 2 columns are combined M/F); WT *n* = 14 (7M/7F), OE *n* = 15 (7M/8F), similarly to total adenine nucleotides (**G**; last 2 columns are combined M/F) WT; *n* = 16 (7M/9F), OE *n* = 15 (8M/7F). There was no difference in the fraction of phosphorylated AMPK protein (**H,I**; last 2 columns are combined M/F), WT *n* = 13 (5M/8F), OE *n* = 15 (5M/10F). Unpaired Student’s *t*-test was used compare WT vs. AK1-OE. Data is mean ± SEM.

Genetic overexpression of AK1 in mouse LV resulted in 31% higher AK1 activity levels in transgenic mice compared to wild-type (*P* = 0.009) (Figure 1A). It is notable that there were no differences in the activity of the rest of AK isoforms expressed by the heart (*P* > 0.05). The above observations demonstrate that AK1 is overexpressed in LV tissue at the level of mRNA, protein and activity.

### Assessment of Compensatory Changes in Energy Metabolism

Creatine kinase (CK) is the major phosphotransfer system in cardiomyocytes, but total CK activity in LV homogenates was not significantly altered in response to AK1-OE (Figure 1B). However, there was a subtle shift in the relative distribution of isoenzyme activities from MM toward MB, therefore CKMM was lower in LV from AK1-OE (*P* = 0.03; Figure 1C). Both males and females showed the same pattern for this isoenzyme shift. AK1-OE mice displayed higher total [Cr] (sum of free creatine and phosphocreatine) levels in LV compared to wild-type littermates (*P* = 0.017; Figure 1D), but in the absence of altered creatine transporter gene expression (Figure 1E). Citrate synthase activity as a marker for total mitochondrial capacity was unchanged by overexpression of AK1 (Figure 1F). An assessment of individual adenine nucleotides is not reliable by HPLC due to rapid interchangeability, but total adenine nucleotide (TAN) pool was unaffected by genotype (*P* = 0.30) (Figure 1G). Western blot of rapidly dissected, snap-frozen, LV detected no differences in total AMPKα protein expression normalised to GAPDH (unpaired *t*-test, *P* = 0.27 data not shown), nor in AMPK phosphorylation at Thr^172^ when normalised to total AMPKα suggesting that AK1 overexpression does not lead to AMPK activation under basal conditions (*P* = 0.85, unpaired *t*-test) (Figures 1H,I).

### ^1^H NMR Metabolomics

**FIGURE 2:**
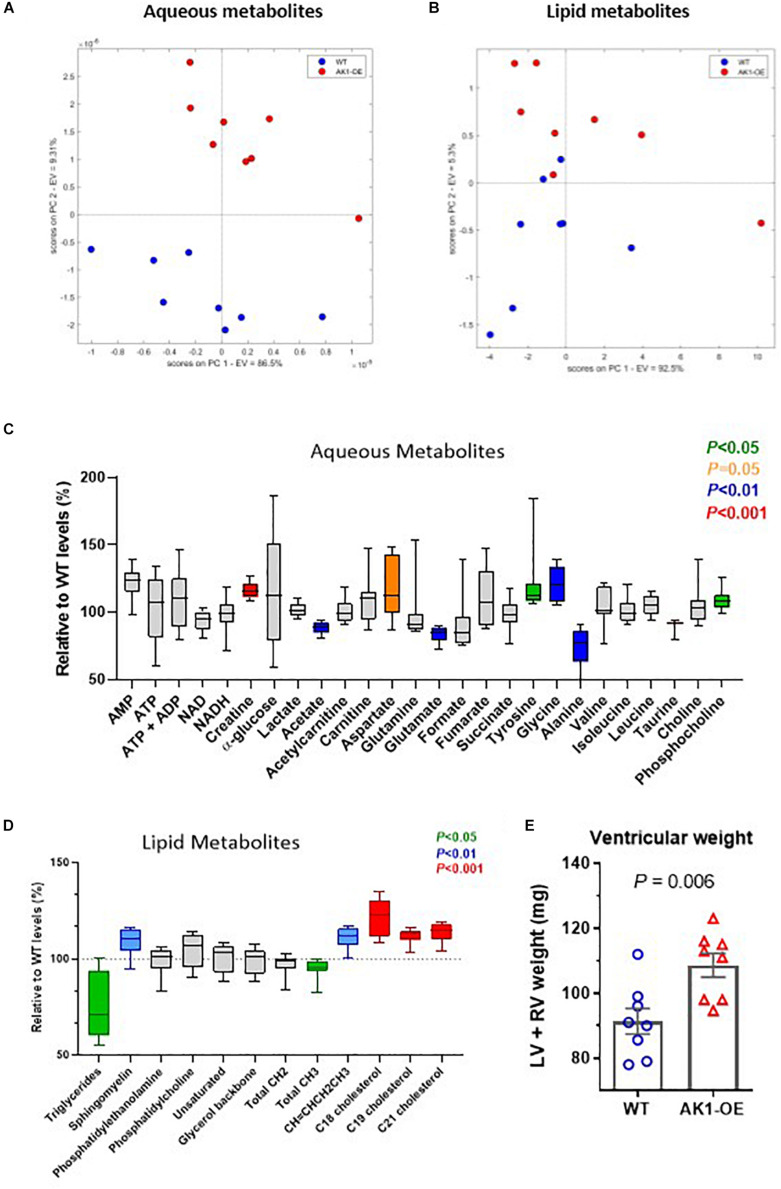
^1^H-MRS metabolomic profile from ventricular myocardium. Principal component analysis (PCA) for aqueous metabolites **(A)** and lipids **(B)**. Concentrations of all detected metabolites are shown relative to mean wild-type (WT) levels. These are represented by box and whiskers plots for aqueous fraction **(C)** and lipid fraction **(D)**. The box indicates median values with 25th and 75th percentiles and the whiskers the min and max values. Significant changes are shown in colour according to legend. Only age-matched males were used, *n* = 8 per genotype, mean age of 25 weeks. Data analysis as described in Online Supplement and by Unpaired Student’s *t*-test. Combined ventricular weight was higher in AK1-OE (AK1/AK1) mice compared to wild-type **(E)**, comparisons by Unpaired Student’s *t*-test.

The unexpected increase in creatine prompted further analysis and measurement of other aqueous and lipid metabolites by ^1^H-NMR in WT and age-matched OE. Unsupervised Principal Component Analysis (PCA) showed efficient separation of the two cohorts (Figures 2A,B). Overexpression of AK1 resulted in a number of unanticipated changes in cardiac metabolite as summarised on Figure 2 and expressed as % vs. WT.

#### Aqueous Metabolites

^1^H NMR confirmed the higher levels of Cr in the AK1-OE LV, in agreement with the HPLC data (Figure 2C, Supplementary Material, and Supplementary Table 2) (*P* < 0.0001). AMP was raised in AK1-OE hearts by 1.2-fold (*P* = 0.05) and increases were also found for aspartate (*P* = 0.051), tyrosine (*P* = 0.045) glycine (*P* = 0.001) and phosphocholine (*P* = 0.01). In contrast, there were lower levels for acetate (*P* = 0.005), glutamate (*P* = 0.001), alanine (*P* = 0.007), and taurine (*P* = 0.0004).

#### Lipid Metabolites

Lipid metabolites such as sphingomyelin, CH = CHCH_2_CH_3_, C18, C19, and C21 cholesterol were higher in OE vs. WT (*P* = 0.009, 0.001 and < 0.001 for C18, C19, and C21, respectively). However, Triglycerides (TG) and total CH_3_ were lower in OE hearts (*P* = 0.039 for both) (Figure 2D, Supplementary Material, and Supplementary Table 3).

### Investigation of LV Morphology

**FIGURE 3:**
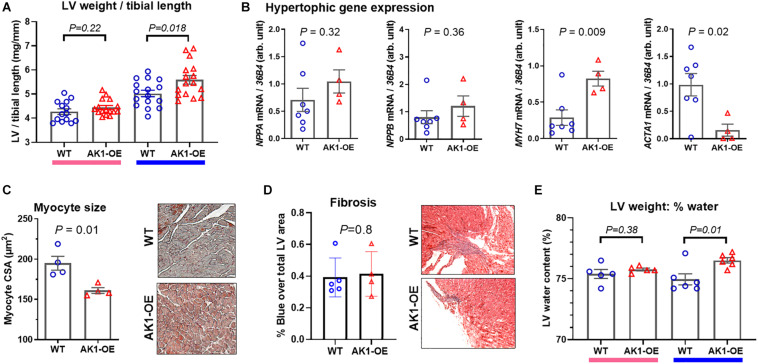
Left ventricular weight and composition in wild-type (WT/WT) and AK1-OE (AK1/AK1) mice. **(A)** LV post mortem weight normalised to tibial length in female mice was not different (left hand columns; WT *n* = 14, OE *n* = 16; Ages 28 ± 4 and 27 ± 4, respectively), while LV/tibial length was higher in male AK1-OE mice (WT *n* = 16, OE *n* = 16 both aged 22 ± 2 weeks). **(B)** This was not associated with systematic activation of foetal hypertrophy genes for atrial natriuretic peptide (*Nppa*), brain natriuretic peptide (*Nppb*), β-myosin heavy chain (*Myh7*) and alpha-skeletal actin (*Acta1*) (WT *n* = 7, OE *n* = 4 all male aged 16–17 weeks). **(C)** LV histological sections (Masson-Goldner Staining) showing average myocyte cross-sectional in WT and AK1-OE male hearts (55–157 myocytes per heart from *n* = 4 hearts) (*P* = 0.01) (20x objective lens). **(D)** Fibrosis in WT transgenic mice, as analysed by MassonGoldner Staining (*P* = 0.8) (10x objective lens). **(E)** LV wet and dry weights were obtained in a subset of mice from panel **(A)** and used to calculate tissue water content, which was elevated only in males (*P* = 0.01). Comparison between two genotypes was by unpaired Student’s *t-*test using Welch’s correction where variances were found to be significantly different.

Despite matching groups for age and male sex, the AK1-OE hearts used for metabolomic analysis were unexpectedly 19% heavier than WT controls: LV + RV weight combined was 109 ± 10 and 91 ± 11 mg, respectively (*P* = 0.006; Figure 2E). This was confirmed in the haemodynamic cohort where male AK1OE mice had higher weights for both LV and RV in absolute terms and when normalised to body weight or tibial length (Figure 3A and Supplementary Table 4). This effect was borderline in females where LV and RV weights were only significantly elevated when normalised to body weight (Supplementary Table 4).

Corroborating data from an independent experimental cohort pre-backcross is included in Supplementary Tables 5, 6.

To determine whether elevated heart weight was due to cardiomyocyte hypertrophy we quantified associated marker genes in male hearts. Gene expression of atrial and brain natriuretic peptides were found to be unaltered by genotype (*P* = 0.32 and 0.36, respectively) while β-myosin heavy chain was elevated (*P* = 0.009) and α-skeletal actin lower (*P* = 0.02) (Figure 3B). This indicates that the wholesale activation of the foetal gene programme associated with hypertrophy is absent in AK1-OE hearts. This was confirmed by histological analysis of myocyte cross-sectional area, which showed that AK1-OE cardiomyocytes were actually smaller than wild-type (*P* = 0.01; Figure 3C), thereby ruling out hypertrophy. Nor were there any differences in the extent of interstitial fibrosis as shown by Masson-Goldner Staining (*P* = 0.8; Figure 3D). Finally, from analysis of LV wet and dry weights it was found that male OE hearts, but not female, had significantly higher water content compared to wild-type (*P* = 0.01; Figure 3E). Taken together this suggests that male AK1-OE have larger hearts than their WT counterparts, but this is not due to cardiomyocyte hypertrophy or fibrosis. Instead this difference is driven by a higher cellular water content. The smaller myocyte CSA probably reflects greater dehydration during tissue processing.

### *In vivo* LV Function

**FIGURE 4:**
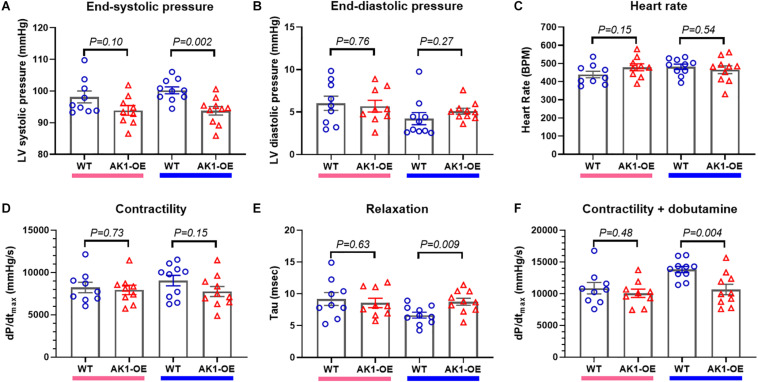
*In vivo* left ventricular haemodynamic measurements. **(A)** There was a trend for LV endsystolic pressure to be lower in AK1-OE (AK1/AK1) females compared to wild-type (left-hand columns) that was statistically significant in the male mice (right-hand columns). LV end-diastolic pressure **(B)**, heart rate **(C)** and contractility (assessed as the maximal rate of pressure change, dP/dtmax; **(D)** were unaffected by genotype for either sex. However, tau, the isovolumetric constant of relaxation was prolonged in male AK1-OE compared to male wild-type **(E)** and the maximum contractility obtained under β-adrenergic stimulation with dobutamine was lower **(F)**. Females *n* = 9 and males *n* = 10 with mean age 17 weeks throughout. Data is mean ± SEM, with same sex comparisons between genotype by Student’s *t-*test.

Baseline haemodynamic function was assessed *in vivo* by LV catheterisation. Male AK1-OE mice had lower end-systolic pressure compared to WT (*P* = 0.002) (Figure 4A), but no significant differences in enddiastolic pressure, heart rate, or contractility as measured by dP/dt_*max*_ (Figures 4B–D). However, tau of isovolumetric relaxation was prolonged in AK1-OE hearts (*P* = 0.009; Figure 4E) and under conditions of maximal β-adrenergic stimulation (dobutamine challenge) the maximum contractility obtained was lower (*P* = 0.004; Figure 4F). No Significant differences were observed in female mice for any of these parameters. Although LV dysfunction in male AK1-OE was subtle, it nevertheless appears to be real, since a similar pattern of dysfunction was observed in preliminary experiments using mice prior to genetic backcrossing (independent supporting data included in Supplementary Tables 5, 6). Cine-MRI in that cohort indicated no differences in either LV volumes or ejection fraction between genotypes under baseline conditions (Supplementary Tables 5, 6).

### Effect of AK1-OE on I/R Functional Recovery

**FIGURE 5:**
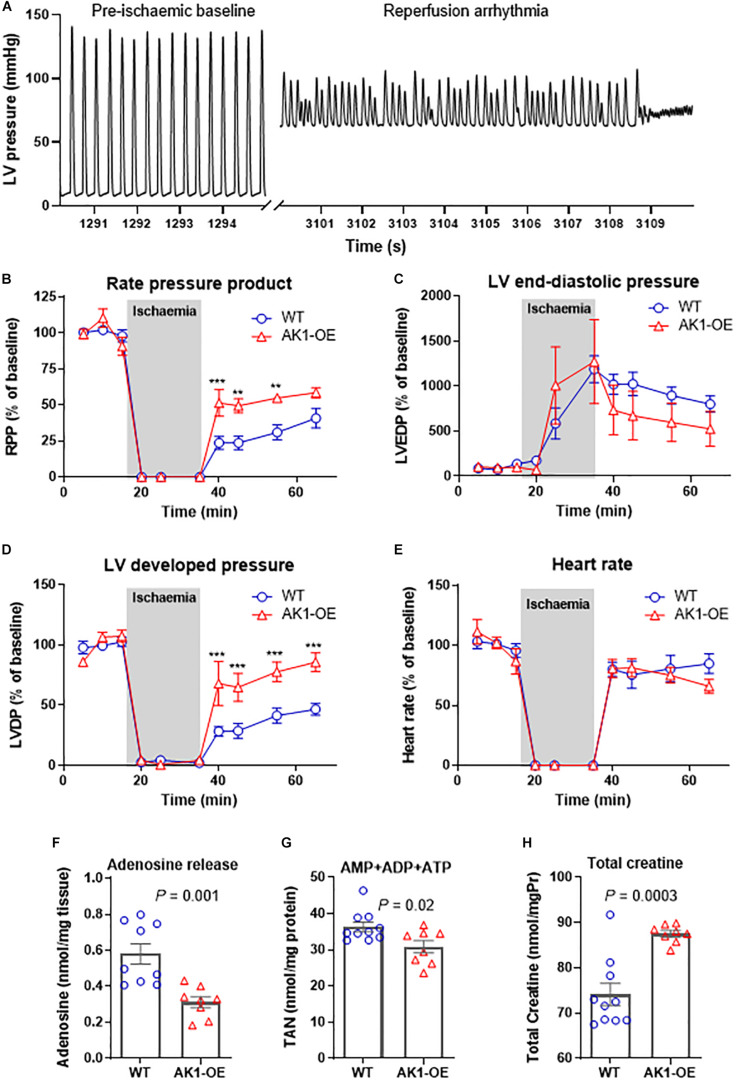
Left ventricular function during global no-flow ischaemia for 20 and 30 min reperfusion in Langendorff perfused hearts. An example pressure trace showing pre-ischaemic baseline function and during terminal reperfusion arrhythmia in an AK1-OE (AK1/AK1) heart **(A)**. Analysis of function in surviving hearts (*n* = 7 wild-type and *n* = 3 AK1-OE) **(B)** showing end-diastolic pressures **(C)**, and developed pressures **(D)**, in addition to heart rate is unaffected **(E)**. Data is mean ± SEM, expressed as a percentage of the average baseline values. Analysis is by two-way ANOVA with Student’s *t*-tests to compare genotypes at all time-points using the Holm-Sidak method to correct for multiple comparisons. In a separate experiment perfusate was collected during the first 10 min of reperfusion for measurement of adenosine release **(F)** and of residual myocardial levels of total adenine nucleotide pool **(G)** and total creatine **(H)**. Data is mean ± SEM (*n* = 9–10 WT and *n* = 8 AK1-OE, all male mice), analysis by two-tailed unpaired Student’s *t*-test.

We then tested whether AK1-OE hearts in Langendorff mode had improved functional recovery from ischaemia. Prolonged bouts of arrhythmia affected most of the AK1-OE hearts, starting during the baseline equilibration period. Originally, a cohort of *n* = 7 AK1-OE and *n* = 7 WT hearts were attempted. Four out of seven AK1-OE hearts developed severe tachycardia and/or fibrillation during reperfusion and did not survive the protocol compared to 0/7 WT hearts (see Figure 5A for representative arrhythmic trace). Despite this, the surviving AK1-OE hearts actually showed better functional recovery than WT. Rate pressure product recovered quicker and to higher values during reperfusion, driven by a trend for lower end-diastolic pressure (Figures 5B,C) that contributed to a robust and sustained increase in developed pressure (Figure 5D), while heart rate was unaffected (Figure 5E).

### Adenosine Release in *ex vivo* Perfusate

To investigate further, a separate cohort of mice were perfused under the same conditions, but with the perfusate collected during the first 10 min of reperfusion. The heart was snap-frozen for biochemical analysis at this point or earlier during reperfusion if it ceased to contract. Functional data at 10 min was obtained from *n* = 5 WT/WT and *n* = 4 AK1/AK1 hearts and was in broad agreement with the previous experiment, with a trend for higher RPP in AK1-OE hearts (5,040 ± 3,625 mmHg.bpm in WT vs. 11,398 ± 8,546, *P* = 0.21).

AK1-OE hearts released significantly less adenosine than wild-type hearts (*P* = 0.001; Figure 5F), and had less residual TAN pool in the myocardium (*P* = 0.02; Figure 5G). Myocardial creatine levels remained significantly higher following reperfusion in the OE hearts (*P* = 0.003; Figure 5H), indicating that changes in metabolite levels are not explained by non-specific differences in cell membrane permeability. This suggests that loss of TAN pool in AK1-OE hearts may pre-dispose to arrhythmia in the *ex vivo* setting.

### Effect of AK1-OE on I/R Injury *in vivo*

**FIGURE 6:**
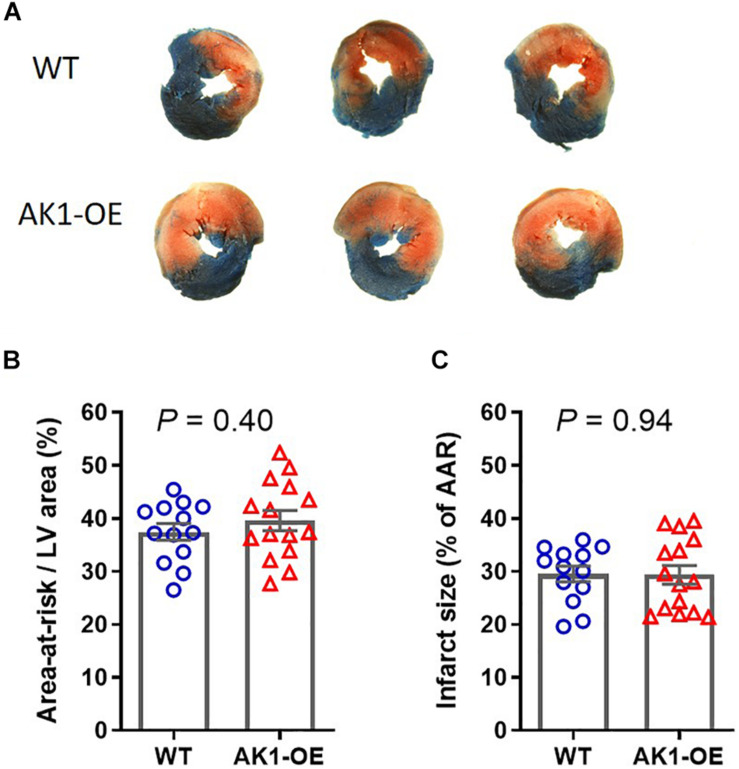
Myocardial infarct size following 45 min regional ischaemia and 24 h reperfusion *in vivo*. **(A)** Example slices from wild-type (WT/WT) and AK1-OE (AK1/AK1) hearts showing typical tricolour staining pattern of blue for unaffected tissue, red + white for the ischaemic area-at-risk, and white indicating areas of tissue necrosis. **(B)** The area-at risk, i.e., the area that was made ischaemic, was not significantly different between genotypes, nor did AK1-OE alter the size of necrotic injury **(C)**. Data is mean ± SEM with comparison by unpaired *t*-test. Wild-type *n* = 13 (6F/7M) and AK1-OE *n* = 15 (8F/7M).

To determine whether this was recapitulated *in vivo*, matching cohorts of WT and AK1-OE mice were subjected to 45 min ischaemia followed by 24 h reperfusion. There was no significant difference in the area-at-risk (AAR; *P* = 0.40), nor in the infarct size expressed as % of AAR (*P* = 0.94) (Figures 6A–C). Notably, none of the mice died of arrhythmia, regardless of genotype. This indicates that elevating AK1 activity in the heart does not influence the extent of I/R injury and that the pro-arrhythmic phenotype is unique to the *ex vivo* setting.

## Discussion

Here, we created a novel transgenic model of cardiomyocyte Adenylate Kinase 1 overexpression to address the hypothesis that this would improve energy homeostasis and thereby cellular survival during metabolic stress, for example, by recruiting the β-phosphoryl group to double the energy provided by ATP (Dzeja et al., 1985). However, no such protection was observed in response to I/R injury, while unexpected consequences were observed in baseline cardiac function and metabolic profile that suggest a subtle role for AK1 in myocardial homeostasis even at rest.

Our cloning strategy made use of Rosa26 targeted integration, which results in a single transgene insertion into a safe chromosomal position (Chen et al., 2011). Hearts from AK1-OE exhibited a desirable gene dosing effect, with homozygotes showing a mild, but quantifiable, elevation in AK1 activity of 31%. While modest, the existence of a baseline phenotype suggests this is physiologically relevant, and we have previously utilised an identical strategy to identify a cardioprotective phenotype in CKmt2-OE mice (Whittington et al., 2018), where enzyme activity was only elevated by 27%.

This also appears to be within the physiological range of endogenous AK1 activation, which is considered compensatory. For example, mice that are deficient in both the muscle and mitochondrial isoforms of creatine kinase (M-CK and CKmt2) have a twofold increase in AK flux compared to wildtype controls (Dzeja et al., 2011). In the context of chronic heart failure, where the CK system is consistently downregulated, AK1 flux is reported to increase by 134%, with the contribution to total ATP turnover rising from 10 to 21% (Dzeja et al., 1999). It is a limitation of our study that we measured maximal enzyme activity rather than AK flux via an ^18^O-assisted NMR approach, which is likely to be a more sensitive measurement since it determines concentrations and metabolic turnover rates of main molecules in energetics and metabolic flux rates (Dzeja et al., 2007a). The technique employs Langendorff perfused hearts, which opens up the possibility of studying energetic flux after I/R. Our current method provides a readout of maximal AK1 activity using spectrophotometric readout and would not require perfusion, which was shown to be problematic for some AK1-OE hearts. Nevertheless, it is reasonable to conclude that the baseline effects observed in AK1-OE hearts are unlikely to reflect direct toxicity due to gross over-expression of transgenic protein. The flip-side, however, is a subtle molecular phenotype that required large experimental groups to resolve.

Our data show lower expression of AK3 protein in LV samples of AK1-OE mice vs. WT, whereas no differences were observed in either AK3 mRNA levels or in remnant AK activity, which represents the combined activity of AK isoforms other than AK1. In contrast, AK2 protein and mRNA remain unaffected. Recent studies in a conditional heart-specific AK2 KO model showed adaptation of AK1, AK3 and AK4 isoenzymes *in vivo*, by a dramatic increase in protein levels vs. WT (Zhang et al., 2021) demonstrating plasticity of the AK system. In general, the dissociation between mRNA and protein levels has been previously reported (Liu et al., 2016).

We observed an unanticipated mild impairment of baseline *in vivo* function. Male AK1-OE had lower LV systolic pressures, prolonged relaxation, and reduced contractile reserve. This was a consistent finding that was observed in two large and independent experimental cohorts, i.e., the main post-backcross study reported herein and the pre-backcross data reported in Supplementary Material. This represents a mild perturbation of normal function since there is no evidence of hypertrophy, elevated LV end-diastolic pressures or lung congestion, and OE hearts maintain a reasonable (though blunted) functional contractile reserve, therefore, these mice patently do not have chronic heart failure.

A number of metabolic changes were unexpectedly observed under baseline conditions. Although AK1-OE hearts had normal total CK activity, there was a redistribution of M-CK from homo-dimer MM to hetero-dimer MB. The physiological significance of this is unknown, but it could alter subcellular localisation, which may be important for direct channelling of ATP to key enzymes (Schlattner et al., 2016). For example, it is notable that B-CK can translocate to the endoplasmic reticulum in response to AMPK phosphorylation (Schlattner et al., 2016).

We also observed a small but robust elevation of total creatine levels in AK1-OE hearts, confirmed in separate samples by both HPLC and ^1^H-NMR. In theory, elevated [Cr] will increase the maximal CK flux capacity (calculated as the product of creatine levels and CK activity (Ingwall and Shen, 2010), which is considered beneficial to energetic status (Lygate et al., 2012; Whittington et al., 2018). Our previous work has shown cardioprotection by elevating [Cr] in the heart (Lygate et al., 2012), but also toxicity at much higher levels (Wallis et al., 2005), however the effect size here is too small to contribute to either. Increased creatine levels usually reflect up-regulation of the creatine transporter (CrT), since this is the unique point of creatine entry into cardiomyocytes and efflux is passive (Zervou et al., 2016a). There is strong post-transcriptional regulation of the CrT, so this can happen in absence of changes in gene expression (ten Hove et al., 2008). Furthermore, AMPK activation by AICAR has been reported to increase creatine-uptake in HL-1 cells and in rat neonatal cardiomyocytes (Darrabie et al., 2011), however this effect has not been described in adult cardiomyocytes and the exact opposite was shown for kidney cells (Li et al., 2010). Nor did we observe activation of AMPK under these basal conditions, despite higher levels of AMP.

Another unexpected phenotype was higher heart weight in male OE vs. WT, suggesting potential LV hypertrophy. However, markers of foetal gene expression were not elevated, pathological fibrosis was absent, and myocyte cross-sectional area was actually smaller in OE hearts. Instead we observed a higher water content in male hearts and for this reason metabolites were normalised to total metabolite spectra rather than heart wet weight to control for differences in tissue H_2_O content. We have previously shown that creatine is a compatible osmolyte (Faller et al., 2018) and we suggest that the higher water may be secondary to the osmotic effect of elevated creatine in AK1-OE hearts. In creatine-deficient mice this results in a compensatory increase in taurine levels (Faller et al., 2018), whereas in mice with elevated creatine levels, due to CrT overexpression, taurine is reduced (Zervou et al., 2016b), as indeed we observe in the current study.

The unexpected effects on creatine led to us examining other metabolomic changes in the AK1-OE heart. Myocardial triglycerides were lower in AK1-OE hearts, which suggests reduced energy storage capacity for aerobic respiration and/or a shift in substrate preference. This could potentially blunt cardiac performance at high workloads as shown when male AK1-OE mice were challenged under conditions of maximal β-adrenergic stimulation with dobutamine infusion. NAD, NADH are unchanged, similarly to succinate and fumarate, which suggests that both redox status and the TCA cycle are unaltered between genotypes. Other metabolite changes are potentially relevant in the setting of injury, but the relevance to basal function is less obvious. Elevated glycine is known to have a cardioprotective effect in I/R injury by delaying opening of the mitochondrial transition pore (Ruiz-Meana et al., 2004), whereas serum levels of tyrosine are prognostic of mortality in patients with acute heart failure (Stryeck et al., 2019) and glutamate and aspartate are preferred myocyte fuel in ischaemia (Arsenian, 1998). Tissue cholesterol levels were higher in AK1-OE and being a membrane component, this could affect cellular integrity during I/R. Indeed, in diabetic perfused hearts high cholesterol changed mitochondrial membrane fluidity in response to I/R *ex vivo* (Ferko et al., 2018). It is a common limitation that these metabolite changes are only a snapshot and do not represent flux through the system. However, they are likely to be downstream of AK1 rather than off-target effects since we used the Rosa26 approach to obtain targeted gene integration and thereby prevent confounding effects from random integration.

Previous studies using AK1 KO mice have indicated that alternative phosphotransfer mechanisms can compensate for AK1 to maintain adenine nucleotides in the absence of metabolic challenge, although to the best of our knowledge, detailed *in vivo* cardiac phenotyping has not been published. However, when exposed to hypoxia AK1-knockout hearts display compromised energetics and signalling, providing direct evidence that AK1 is essential in maintaining homeostasis during metabolic stress (Pucar et al., 2000). AK1-KO showed adaptation including sustained creatine kinase flux, increased glucose-6-phosphate post-ischaemia, and 40% of β-phosphoryl turnover was maintained, suggesting that other AK isoforms can compensate in this scenario (Pucar et al., 2002). The effect on cell survival and I/R injury were not determined, nor is it known whether increasing AK1 activity may be protective.

We therefore tested functional recovery following I/R *ex vivo* and found AK1 elevation to be a double-edged sword. AK1-OE hearts did indeed have an improved recovery in contractile function, but many OE hearts did not complete the protocol due to the development of terminal arrhythmias. Perfused heart is a very pro-arrhythmic setting, as is reperfusion (Bernier et al., 1986), so even small differences may be sufficient to push the heart over the threshold to lethal arrhythmia. In contrast, arrhythmias were not observed during our *in vivo* I/R protocol and there was no effect of AK1-OE on infarct size at 24 h, indicating that cell survival was unaffected. It should be noted that the perfused heart model reports on the initial 30 min of recovery and does not necessarily reflect subsequent cell loss due to commitment to cell death pathways, therefore it is not necessarily linked to longer term cell survival at 24 h.

AK1 activity promotes cellular adenosine release under ischaemic conditions and this is blunted in KO hearts (Pucar et al., 2000). Adenosine peaks in the first 3 min of reperfusion (Kin et al., 2005) and is cardioprotective via downstream paracrine effects (Heusch, 2010), but this may also have deleterious effects on cellular energetics due to consequent loss of total adenine nucleotides. However, when tested in Langendorff mode, adenosine release during the first 10 min of reperfusion was significantly lower from AK1-OE hearts compared to WT. Counter-intuitively, residual myocardial TAN pool was also lower in AK1-OE. Since we know the starting TAN pool is not different, this suggests that AK1-OE hearts lose adenosine more rapidly during the 2–3 min cannulation period, consequently leading to lower residual TAN pool and energetic compromise that begets arrhythmia. We cannot rule out that the beneficial effect on function in the surviving OE hearts may reflect survival bias and low n numbers. It is a limitation that we did not measure other downstream products of purine degradation, e.g., xanthine and hypoxanthine (Ingwall and Shen, 2010). These are substrates for cellular xanthine oxidases that could result in generation of ROS and may contribute to arrhythmia/functional recovery. However, this is not of physiological relevance since the effects we observed are clearly an artifact of the perfused heart preparation and were not observed *in vivo*.

With AK only accounting for ∼10% of phosphotransfer under resting basal conditions, there is a tendency to consider the AK system as an example of metabolic redundancy, which only gains physiological importance when other phosphotransfer systems fail. In this study it is notable that we did not observe a consistent cardioprotective effect of AK1 under such conditions, but an unexpected subtle baseline phenotype emerged. This suggests that AK1 has a broad role in fine-tuning basal energy homeostasis, most likely due to the ability to rebalance local concentrations of adenine nucleotides.

## Data Availability Statement

The original contributions presented in the study are included in the article/Supplementary Material, further inquiries can be directed to the corresponding author/s.

## Ethics Statement

The animal study was reviewed and approved by the Committee for Animal Care and Ethical Review at the University of Oxford.

## Author Contributions

CL and SZ: conceptualisation and writing—original draft preparation. SZ, DM, HW, HL, KC, KP, PO, TE, and JS: methodology. DM, SZ, HL, HW, KC, KP, PO, and TE: formal analysis and investigation. CL, SZ, DM, HL, KC, KP, PO, TE, JS, and SN: writing—review and editing. CL, SN, and JS: funding acquisition. SZ, JS, SN, and CL: supervision. All authors contributed to the article and approved the submitted version.

## Conflict of Interest

The authors declare that the research was conducted in the absence of any commercial or financial relationships that could be construed as a potential conflict of interest.
