# Supplementary material for: Subtle Role for Adenylate Kinase 1 in Maintaining Normal Basal Contractile Function and Metabolism in the Murine Heart
Source: Front Physiol. 2021 Mar 31;12:623969. doi: 10.3389/fphys.2021.623969 (PMC8044416; doi:10.3389/fphys.2021.623969)
Supplement: Supplementary file 1 [file Data_Sheet_1.docx]

Supplementary Material

# Supplementary Methods

**1.1 Generation and breeding of transgenic mice**

Mouse AK1 open reading frame was amplified by PCR from mouse LV cDNA using Phusion polymerase (NEB) and cloned into a vector containing the αMHC promoter (1) incorporating a *Sal*I and Kozak sequence at the 5’ end/ N-terminus and a C-terminal HA tag/stop sequence/*Hind*III restriction site at the 3’ end. The αMHC vector and AK1-HA fragment were ligated using T4 ligase (NEB), and the ligation product was cloned into *E. coli* by selecting ampicillin-resistant colonies before purifying DNA in the form of Mini and Maxi preps (Qiagen). Sequencing of the resulting cloned αMHC-AK1-HA plasmids was outsourced to Source BioScience (Oxford) and were analysed using 4Peaks (A. Griekspoor and Tom Groothuis, mekentosj.com)(2). Consensus sequences were constructed from the individual sequencing reads using Serial Cloner, and subsequently checked for alignment against the predicted sequence (**Suppl. Fig. 1A**).

αMHC-AK1-HA-polyA was cloned into a vector containing the machinery for PhiC31 integrase mediated cassette exchange at the Rosa26 locus as described before, by Core Transgenics group, WCHG, University of Oxford (2) (**Suppl. Fig. 1B**). The exchange vector was co-electroporated with an expression cassette for PhiC31 into acceptor embryonic stem cells. Transgenic mice were generated by microinjecting embryonic stem cells into C57BL/6J blastocysts, which were implanted into pseudo-pregnant females to create chimeric founder mice. Male chimeric founders were mated with wild-type C57BL/6J females (Envigo, Huntingdon, UK). Homozygous transgenic (AK1/AK1) mice were generated by breeding F1 WT/AK1 males with F1 WT/AK1 females and back-crossed onto C57BL/6JOlaHsd genetic background for 10 generations. Transgenic pups were identified by PCR (**Suppl. Fig. 1C**).

**1.2 Genomic DNA extraction and genotyping**

DNA was extracted from mouse ear biopsies following enzymatic digestion in 500 µL lysis buffer (100 mM EDTA, 50 mM Tris-HCl (pH 8.0), 1% SDS, 200 mM NaCl), supplemented with 3.3 µL Proteinase K (20 mg/mL), before overnight incubation and purification by isopropanol/ ethanol precipitation as described before (2). Air-dried pellets were re-suspended in 300 µL T0.1E buffer (10mM Tris, 0.1mM EDTA pH 8.0) and PCR reactions were performed using oligonucleotides Rosa26_F and _R, in addition to (AK1_F and AK1_R) to detect Rosa26 and to determine zygosity. All reactions were carried out using Immolase DNA polymerase (Bioline, SLS, Nottingham UK) and either DNA Engine, MJ Research Peltier Thermal Cycler or Applied Biosystems Life Technologies Thermal Cycler, as described in (2).

**1.3 AK1 activity assays**. Homogenised tissue (5-10 mg) was added to 1 mL chilled extraction buffer (150 mM NaCl, 60 mM Tris-HCl, 5 mM EDTA, 0.2% Titron-X-100, 1x Roche cOmplete Protease inhibitor tablet per 10 mL, pH 7.5) and homogenised using a polytron PT1200. The resulting suspension was centrifuged (10,000 rpm, 10 mins, 4 °C). A BCA assay was performed to determine protein concentration. Assay mixture (100 mM potassium acetate, 20 mM HEPES, 20 mM glucose, 4 mM MgCl_2_, 2 mM NADP^+^, 1 mM EDTA) was warmed to 30 °C.

To measure total AK activity, the assay mixture (1.746 mL), hexokinase (Merck Sigma) (18 µL) and G-6-P dehydrogenase (Merck Sigma) (16 µL) were added to 20 µL of sample in a cuvette. ADP (200 µL of 20 mM stock at pH 7.5 for 2 mM final concentration) was added to initiate the reaction. Phosphate buffer constituted of K_2_HPO_4_ (71.7 mL of 1M stock for 71.7 mM final concentration), KH_2_PO_4_ (28.3 mL of 1M stock for 28.3 mM final concentration) in 900 mL H_2_O. The spectrophotometer was run with the following parameters: lag time = 0, duration = 8 min, period = 20s, temp = 30 °C, Absorbance at 340 nm.

Samples were incubated with DTNB (5,5-dithio-bis-(2-nitrobenzoic acid) (5 µL of 5 mM stock in phosphate buffer for 0.5 mM final concentration) for 15 minutes at room temperature before measuring activity as above. DTNB inhibits AK-1 activity, so this measurement provides the value for remnant, AK-2 and AK-3 activities. Both total AK and AK1 activities (µmol/min/mg) were calculated using the slopes generated from the spectrophotometer via the following formula: = Activity (µmol/min/mg) = (slope*0.161)*(total assay volume/sample volume)/protein (mg ml^-1^). AK-1 activity was calculated by subtracting the DTNB-inhibited activity from the total activity.

**1.4 RNA extraction and qRT-PCR.** For testing transgenic, total and endogenous mRNA levels, a cohort of n=8 mice per group were analysed. In particular, WT/WT 4M/4F (av. age 7.36); WT/AK1 5M/3F (av. age 8.36 weeks); AK1/AK1 5M/3F (av. age 7.64 weeks). For oligonucleotides please see **Suppl. Table 1**. LV samples were separated from RV and snap-frozen in liquid N_2_, then crushed on dry ice. Total RNA was extracted from ~ 10mg left ventricular frozen powdered tissue using phenol/chloroform extraction (Trizol reagent; Invitrogen) and further purified using RNeasy Mini Kit (Qiagen). Real-time PCR was performed using 1ng/μl total RNA as input, iScript™ One-Step RT-PCR Kit With SYBR® Green and a CFX96 machine (Bio-Rad). The levels of expression of all transcripts were normalized to 36B4 using the 2^-∆∆CT^ method and expressed as percentage of controls (3). Hypertrophy markers were tested using previously reported oligo sequences (**Suppl.** **Table 1**)(4, 5). For this purpose, WT and AK1-OE were analysed as follows, at average age 16.5 weeks; for ANP: 7WT vs 4OE; for BNP: 8WT vs 4OE; for βMHC: 7WT vs 4OE; for αSA: 4WT vs 4OE.

The lack of a pronounced increase in AK1 protein and activity was further investigated by testing whether overexpression of αMHC-AK1 led to downregulation of endogenous AK1 transcript. For this purpose, allele–specific qRT–PCR was performed to quantify expression of transgenic, endogenous, and total AK1 mRNA. In order to specifically amplify endogenous AK1 mRNA, the exon–spanning sense primer previously used for detection of transgenic mRNA (Endog exon 5_6 AK1) was paired with an antisense primer designed to bind in the 3’ untranslated region, which is not present in the transgenic sequence (Rev AK1 endog UTR; **Suppl.** **Table 1**). Total mRNA was measured using a pair of primers which bind within the coding sequence, and therefore amplify both the endogenous and transgenic transcripts.

**1.5 AK protein expression and AMPK phosphorylation levels.** Total protein was extracted from snap-frozen LV samples by homogenising in ice old RIPA buffer (Merck Sigma) containing cOmplete^™^ Protease Inhibitor Cocktail (Roche) according to manufacturer’s instructions (Roche) and 10mM DTT (2). After quantification using a BCA protein assay (Thermo), denaturing using 1x Reduction and 1x LDS buffers (Invitrogen; according to manufacturer’s protocol) and boiling at 95^o^C for 5 minutes, 50μg protein was analysed on precast mini protean gels (Bio-Rad) and blotted onto PVDF by using a semi-dry Bio-Rad transblot system. The membranes were probed against an anti-AK1 antibody (Santa Cruz biotech #sc-365316) or anti-AK2 (H-65, Santa Cruz Biotech #sc-28786) or anti-AK3 (H-45, Santa Cruz Biotech #sc-28787) and for normalisation purposes stripped and re-probed against β-tubulin (abcam #ab6046). Protein bands were captured and analysed using Image lab (Bio-Rad) quantification tools and normalised over the band intensity values for β-tubulin.

For determining AMPK phosphorylation levels, LV was rapidly removed and freeze-clamped while still beating and placed in liquid N_2_. Protein was extracted as above using RIPA buffer with additional phoSTOP phosphatase inhibitors (Roche; according to manufacturer’s instructions). Protein blots were analysed as described before (6) using primary antibodies (Cell Signaling Technologies) against total AMPKα and phospho AMPKα (antibody detects endogenous AMPKα only when phosphorylated at Threonine^172^). Following quantification of chemiluminescent signal using Chemi-Doc imaging system and Image Lab software (Bio-Rad), AMPK activation was calculated by the ratio of phospho/total AMPKα expression.

**1.6 Biochemical phenotyping**

**1.6.1 Creatine kinase.** Samples were processed as reported previously (2). Briefly, 5mg of frozen powdered LV tissue was homogenised in 1mL of ‘ice buffer’ (0.08mM K_2_HPO4, 1mM EGTA, 0.02mM KH_2_PO_4_, and 1mM β-mercaptoethanol)(all solutions were from Merck Sigma). Samples were vortexed and 150µL aliquots removed for determination of protein by the Lowry method. To the remaining sample, a final concentration of 0.1% v/v Triton X-100 was added to permeabilise cells. Samples were stored on ice for at least 30 minutes to allow precipitation of salts and cellular debris and the supernatant was used for all enzyme activity measurements.

**1.6.2 Total creatine kinase activity**. Twenty (20) µL of 1:5 (in ‘ice buffer’) diluted sample supernatant was incubated with 1mL of CK-NAC reagent (Thermo Fisher Scientific) at 30°C. After a 3-minute lag time, CK activity was quantified spectrophotometrically by measuring the increase in absorbance at 340nm over 2 minutes, as a result of NADH production. The assay was performed in triplicate and results normalised to protein concentration.

**1.6.3 Activity of creatine kinase isoenzymes**. Sample supernatant was diluted 1:20 in ‘ice buffer’ and incubated with 1% CK isoenzyme activator for 10 minutes prior to use. Creatine kinase isoenzymes were separated according to their electrophoretic mobility on an agarose gel, followed by incubation with CK isoenzyme chromogen, which allowed for visualisation of the bands. Relative activities of individual CK isoenzymes were quantified by densitometry. All reagents were provided within the SAS-1 CK VIS-12 Isoenzyme kit (Helena Biosciences). Absolute activities for each isoenzyme were calculated by multiplying relative isoenzyme activity by total CK activity.

**1.6.4 Citrate synthase**. Fifty (50) µL of sample supernatant was incubated with 850μL of reaction mixture (0.35mM acetyl-CoA, 0.12mM DTNB) at 25°C. After 3 minutes, 100μl of 1mM oxaloacetate was added into the reaction mixture. Citrate synthase activity was immediately assessed spectrophotometrically by measuring the increase in absorbance at 412nm over 1 minute, as a result of 5-thio-2-nitrobenzoate (TNB^2-^) production. The assay was performed in duplicate and results normalised to protein concentration.

**1.6.5 Tissue histology**. All samples were harvested and fixed in 10% neutral buffered formalin (Shandon, UK) and then processed by dehydration in graded concentrations of ethanol, cleared in Histo-Clear and infiltrated with paraffin wax, prior to embedding into paraffin wax blocks using an automated tissue processor (Histomaster, Bavimed). Cross-sections of 5 µm thickness were collected throughout the LV and mounted on Superfrost PLUS (Solmedia) slides for staining and analysis. Dewaxing of paraffin sections was performed for 2 hr at 60^o^C before staining with Masson-Goldner Staining Kit (Merck Millipore).

Slides were stained with Masson-Goldner Trichrome and prepared for light microscopy, were imaged using QCapture Pro 7 (Teledyne QImaging Inc.) for fibrosis, (10x objective lens, 10x eyepiece = 100 x overall magnification) and for CSA (QImaging, 20x objective lens, 10x eyepiece = 200 x overall magnification).

**1.6.6 Cine-MRI methods**. Mice were anaesthetised with isoflurane 1.5–2% in oxygen with ECG and respiration monitoring to ensure depth of anaesthesia and homeothermic temperature control as described before (2). Imaging was carried out on a 9.4T (400MHz) MR system (Agilent Technologies) and global cardiac functional parameters were measured as previously reported (7).

**1.6.7 Tissue harvest and dual-phase extraction of metabolites.** ^1^H NMR metabolomics adapted from Whittington *et al*., 2018 (2). In particular, combined left and right ventricle were removed from WT and age matched n = 8 AK1-OE and freeze-clamped using Wollenberger tongs in liquid N_2_. Frozen tissue samples were crushed, and wet weight recorded. A spatula of heart tissue was dissolved in 2mL each of iced methanol, chloroform, and ultra-pure water and vortexed. Samples were centrifuged for 1 hour at 3600 rpm at 4°C to separate aqueous, protein and lipid layers. The aqueous layer was further purified with Chelex 100 (Merck Sigma) through an additional 3,600 rpm centrifugation step at 4^o^C. The aqueous component was then mixed with 15µL of universal pH indicator solution (Merck Sigma) and stored at -80^o^C. The lipid layer was placed into a glass scintillation vial and left to air dry naturally over several hours in a fume cupboard at room temperature and then stored at -80^o^C until analysis.

**1.6.8 NMR analysis of metabolite samples.** Samples were analysed using a vertical-bore, Ascend ultra-shielded Bruker 14.1 T (600 MHz) spectrometer with a Neo console and a nitrogen-cooled TCI Prodigy probe at 298K. Freeze-dried aqueous extracts were re-dissolved in 600µL deuterated water (D_2_O containing 8 g/L NaCl, 0.2 g/L KCl, 1.15 g/L Na_2_HPO_4_, 0.2 g/L KH_2_PO_4_ and 0.0075% w/v trimethylsilyl propanoic acid, TSP) and the pH was adjusted to 7 using 1M HCl or 1M NaOH where necessary. Dried lipid extracts were reconstituted in 600µL deuterated chloroform (CDCl_3_) containing 0.05% v/v tetramethylsilane (TMS). For aqueous samples, a NOESY 1D pulse sequence was used, with 64 scans, 4 dummy scans and 20.8 ppm sweep width, a repetition time of 4s per scan, 90° flip angle and experiment duration of 7 minutes. For lipid samples, a ZG pulse sequence was used, with 64 scans, 4 dummy scans and 19.8 ppm sweep width, a repetition time of 3s per scan, 90° flip angle and experiment duration of 6.5 minutes. TopSpin 4.0 software was used for data acquisition and for metabolite quantification. Assignment of metabolites to their respective peaks was carried out based on previously obtained data, confirmed by chemical shift using Chenomx and referenced to published data (8, 9).

Metabolite signals were quantified by normalising to the total spectrum area (after removing impurities like methanol) corresponding to total metabolite content, expressed in arbitrary units.

**1.6.9 Metabolomics Data Analysis**. Mean metabolite concentrations of transgenic (T) and wild type control (C) groups are expressed as % (T/C). The propagated standard error (SEM) of the ratio was calculated using the formula (𝑇/𝐶)=(𝑇/𝐶)√(𝑆𝐸𝑇/𝑇)^2^+(𝑆𝐸𝐶/𝐶)^2^, assuming the covariance between the two groups is zero, i.e. C and T are uncorrelated.

# Supplementary Tables

| **Cloning** |  |
| --- | --- |
| AK1_F_SalI/Kozak | 5’GGGGGTCGACACCATGGCCAGTGCCTTCTC3’ |
| AK1_R_HA/**stop**/HindIII | 5’ggggaagct**tca**tgcgtaatctggaacatcgtatgggta3’ |
| **Genotyping** |  |
| AK1_F | 5’CCTCAGGCACCCTTACCCCACAT3’ |
| AK1_R | 5’CCACCACAAAGATGATCTTGGCC3’ |
| Rosa26_F | 5’ATACCTTTCTGGGAGTTCTCTGCTGC3’ |
| Rosa26_R | 5’GGAGCGGGAGAAATGGATATG3’ |
| **qRT-PCR** |  |
| Transgenic sense | 5’CCTCAGGCACCCTTACCCCACAT3’ |
| Transgenic anti-sense | 5’ CCACCACAAAGATGATCTTGGCC3’ |
| Endog exon 5_6 AK1 | 5’GTGCGCAAGGTCAATGCCG3’ |
| Rev AK1 endog UTR | 5’GGGAGCTGGCCAAGGGATC3’ |
| Total sense | 5’CCAATGGCTTCCTGATCGA3’ |
| Total anti-sense | 5’GCAGTGTGGGCTGTCCAAT3’ |
| AK2 sense | 5’GTGTCTGTCATTTGGCCAC3’ |
| AK2 anti-sense | 5’CGTCACTCACCAGTTTCCC3’ |
| AK3 sense | 5’GCAGGGCACAGAAATCGGT3’ |
| AK3 anti-sense | 5’GCTACACTGGGTAAGGGTT3’ |
| 36B4 sense | 5’AGATTCGGGATATGCTGTTGG3’ |
| 36B4 antisense | 5’TCGGGTCCTAGACCAGTGTTC3’ |
| **Hypertrophy foetal markers** |  |
| ANP sense | 5’GTGTACAGTGCGGTGTCCAA3’ |
| ANP antisense | 5’ACCTCATCTTCTACCGGCATC3’ |
| BNP sense | 5’GAGGTCACTCCTATCCTCTGG3’ |
| BNP antisense | 5’GCCATTTCCTCCGACTTTTCTC3’ |
| β-MHC | 5’GCATTCTCCTGCTGTTTCCTT3’ |
| β-MHC antisense | 5’TGGATTCTCAAACGTGTCTAGTGA3’ |
| α-skeletal actin sense | 5’GTACCACCGGCATCGTGTT3’ |
| α-skeletal actin antisense | 5’GTGAGGTCGCGACCGGCCA3’ |

**Supplementary Table 1**. Oligonucleotides used for generation and phenotyping of AK1-OE mouse model and qRT-PCR gene expression studies.

|  | **WT (*n*=8)**  **(arbitrary units)** | **WT**  **SEM** | **AK1-OE (*n*=8)**  **(Arbitrary units)** | **AK1-OE**  **SEM** | ***P* value**  **(*t* test)** |
| --- | --- | --- | --- | --- | --- |
| **AMP** | 2.008 | 0.177 | 2.450 | 0.080 | 0.052 |
| **ATP** | 4.316 | 0.217 | 4.438 | 0.359 | 0.789 |
| **NAD** | 1.330 | 0.043 | 1.245 | 0.034 | 0.166 |
| **NADH** | 0.454 | 0.044 | 0.444 | 0.021 | 0.851 |
| **Creatine** | 63.545 | 0.561 | 74.108 | 1.263 | **<0.001** |
| **α-glucose** | 0.625 | 0.028 | 0.739 | 0.088 | 0.266 |
| **Lactate** | 52.665 | 1.722 | 53.654 | 0.917 | 0.643 |
| **Acetate** | 2.214 | 0.064 | 1.954 | 0.033 | **0.005** |
| **Acetyl carnitine** | 10.827 | 0.506 | 10.880 | 0.330 | 0.936 |
| **Carnitine** | 9.446 | 0.589 | 10.332 | 0.611 | 0.364 |
| **Aspartate** | 4.483 | 0.121 | 5.319 | 0.346 | **0.051** |
| **Glutamine** | 27.511 | 0.649 | 27.164 | 2.054 | 0.883 |
| **Glutamate** | 21.863 | 0.695 | 18.272 | 0.419 | **0.001** |
| **Formate** | 0.376 | 0.013 | 0.344 | 0.027 | 0.327 |
| **Fumarate** | 0.219 | 0.021 | 0.241 | 0.016 | 0.445 |
| **Succinate** | 7.641 | 0.432 | 7.510 | 0.302 | 0.820 |
| **Tyrosine** | 0.445 | 0.013 | 0.540 | 0.038 | **0.045** |
| **Glycine** | 2.347 | 0.046 | 2.831 | 0.101 | **0.001** |
| **Alanine** | 11.479 | 0.685 | 8.535 | 0.526 | **0.007** |
| **Valine** | 0.664 | 0.034 | 0.691 | 0.032 | 0.602 |
| **Isoleucine** | 0.793 | 0.026 | 0.804 | 0.027 | 0.782 |
| **Leucine** | 2.213 | 0.051 | 2.332 | 0.056 | 0.161 |
| **Taurine** | 158.787 | 0.982 | 144.032 | 2.397 | **<0.001** |
| **Choline** | 1.769 | 0.054 | 1.861 | 0.089 | 0.421 |
| **Phosphocholine** | 5.264 | 0.066 | 5.758 | 0.141 | **0.010** |

**Supplementary Table 2.** Relative abundance of aqueous metabolites normalised

over total metabolites and shown as mean ± SEM for WT and OE (n=8 each, males).

|  | **WT (*n*=8)**  **(arbitrary units)** | **WT (SEM)** | **AK1-OE (*n*=8)**  **(arbitrary units)** | **AK1-OE(SEM)** | ***P* value (*t* test)** |
| --- | --- | --- | --- | --- | --- |
| **Triglycerides** | 1.162 | 0.095 | 0.877 | 0.068 | **0.039** |
| **Sphingomyelin** | 0.906 | 0.010 | 0.984 | 0.022 | **0.009** |
| **Phosphatidylethanolamine** | 5.360 | 0.065 | 5.303 | 0.132 | 0.722 |
| **Phosphatidylcholine** | 31.469 | 0.331 | 32.841 | 0.937 | 0.218 |
| **Unsaturated** | 65.398 | 0.746 | 65.734 | 1.612 | 0.862 |
| **Glycerol backbone** | 6.560 | 0.091 | 6.497 | 0.151 | 0.742 |
| **Total CH_2_** | 308.685 | 2.911 | 299.906 | 6.139 | 0.247 |
| **Total CH_3_** | 53.345 | 0.593 | 50.591 | 0.962 | **0.039** |
| **CH=CHCH_2_CH_3_** | 10.416 | 0.169 | 11.556 | 0.198 | **0.001** |
| **C18 cholesterol** | 2.170 | 0.028 | 2.641 | 0.071 | **<0.001** |
| **C19 cholesterol** | 3.972 | 0.060 | 4.441 | 0.054 | **<0.001** |
| **C21 cholesterol** | 3.606 | 0.035 | 4.108 | 0.058 | **<0.001** |

**Supplementary Table 3.** Relative abundance of lipid metabolites normalised over total metabolites and shown as mean ± SEM for WT and OE (n=8 each, males).

| **Post-backcross** | **WT** | | **AK1-OE** | *P* value |
| --- | --- | --- | --- | --- |
| *Females* | *n=14* | | *n=16* |  |
| Age (weeks) | 28 ± 4 | | 27 ± 4 | 0.81 |
| Body weight (g) | 24.8 ± 0.5 | | 23.9 ± 0.5 | 0.19 |
| LV weight (mg) | 78 ± 2 | | 81 ± 2 | 0.27 |
| LV / body weight (mg/g) | 3.13 ± 0.09 | | 3.38 ± 0.07 | **0.03** |
| LV / tibial length (mg/mm) | 4.27 ± 0.11 | | 4.45 ± 0.08 | 0.22 |
| RV weight (mg) | 22 ± 0.6 | | 23 ± 0.7 | 0.24 |
| RV / body weight (mg/g) | 0.88 ± 0.03 | | 0.96 ± 0.02 | 0.03 |
| RV / tibial length (mg/mm) | 1.20 ± 0.03 | | 1.27 ± 0.04 | 0.20 |
| *Males* | | *n=16* | *n=16* |  |
| Age (weeks) | | 22 ± 2 | 22 ± 2 | 1.00 |
| Body weight (g) | | 31.7 ± 0.7 | 31.2 ± 0.8 | 0.61 |
| LV weight (mg) | | 94 ± 3 | 104 ± 3 | **0.02** |
| LV / body weight (mg/g) | | 2.98 ± 0.11 | 3.36 ± 0.13 | **0.03** |
| LV / tibial length (mg/mm) | | 5.02 ± 0.14 | 5.59 ± 0.18 | **0.02** |
| RV weight (mg) | | 26 ± 1 | 31 ± 2 | **0.01** |
| RV / body weight (mg/g) | | 0.83 ± 0.03 | 1.00 ± 0.06 | **0.01** |
| RV / tibial length (mg/mm) | | 1.41 ± 0.04 | 1.66 ± 0.08 | **0.01** |

**Supplementary Table 4**. Post-mortem ventricular weights in male and female mice after backcrossing to C57BL/6J. Data are mean ± SEM with inter-group comparison by Student’s t-test.

| **Male pre-backcross** | **WT**  (n=12) | **AK1-OE**  (n=13) | *P* value |
| --- | --- | --- | --- |
| Age (weeks) | 15.0 ± 0.2 | 15.3 ± 0.2 | 0.39 |
| Body weight (g) | 33.9 ± 1.0 | 32.9 ± 0.8 | 0.42 |
| LV weight (mg) | 98 ± 3 | 105 ± 2 | **0.04** |
| LV / tibial length (mg/mm) | 5.2 ± 0.1 | 5.6 ± 0.1 | **0.03** |
|  |  |  |  |
| Heart rate (BPM) | 475 ± 13 | 489 ± 12 | 0.43 |
| End-systolic pressure (mmHg) | 103 ± 2 | 97 ± 3 | 0.15 |
| End-diastolic pressure (mmHg) | 4.1 ± 0.8 | 6.2 ± 0.9 | 0.11 |
| dP/dt_max_ (mmHg/s) | 9150 ± 628 | 7543 ± 622 | 0.07 |
| dP/dt_min_ (mmHg/s) | -8642 ± 790 | -5980 ± 594 | **0.01** |
| Tau (ms) | 7.2 ± 0.7 | 9.8 ± 1.0 | **0.04** |
| *+ dobutamine* |  |  |  |
| Heart rate (BPM) | 580 ± 12 | 572 ± 18 | 0.73 |
| dP/dtmax (mmHg/s) | 14326 ± 847 | 9938 ± 891 | **0.002** |
| dP/dtmin (mmHg/s) | -9626 ± 610 | -7738 ± 727 | 0.06 |
| Tau (ms) | 5.9 ± 0.4 | 7.5 ± 0.8 | 0.08 |
|  |  |  |  |
| *Cine MRI* | (n=3) | (n=4) |  |
| Age (weeks) | 14.9 ± 0.3 | 14.3 ± 0.4 | 0.33 |
| Body weight (g) | 31.4 ± 1.9 | 31.2 ± 1.2 | 0.94 |
| LV mass (mg) | 91 ± 1 | 102 ± 4 | 0.08 |
| Heart rate (bpm) | 425 ± 47 | 416 ± 37 | 0.88 |
| End-diastolic volume (µL) | 64 ± 5 | 61 ± 3 | 0.64 |
| End-systolic volume (µL) | 31 ± 2 | 35 ± 3 | 0.36 |
| Ejection fraction (%) | 52 ± 1 | 42 ± 5 | 0.15 |
| Stroke volume (µL) | 33 ± 3 | 26 ± 4 | 0.23 |
| Cardiac output (mL/min) | 13.9 ± 2.2 | 11.1 ± 2.4 | 0.44 |

**Supplementary Table 5.** Left ventricular haemodynamics and MRI parameters in male mice prior to backcrossing. Data are mean ± SEM with inter-group comparison by Student’s t-test.

| **Female pre-backcross** | **WT**  (n=8) | | **AK1-OE**  (n=13) | *P* value |
| --- | --- | --- | --- | --- |
| Age (weeks) | 15.2 ± 0.4 | | 15.4 ± 0.3 | 0.72 |
| Body weight (g) | 25.7 ± 1.2 | | 24.4 ± 1.0 | 0.42 |
| LV weight (mg) | 78 ± 2 | | 79 ± 2 | 0.92 |
| LV / tibial length (mg/mm) | 4.2 ± 0.1 | | 4.3 ± 0.1 | 0.86 |
|  |  | |  |  |
| Heart rate (BPM) | 466 ± 30 | | 439 ± 17 | 0.40 |
| End-systolic pressure (mmHg) | 101 ± 3 | | 91 ± 4 | 0.06 |
| End-diastolic pressure (mmHg) | 5.7 ± 1.5 | | 6.6 ± 1.4 | 0.66 |
| dP/dt_max_ (mmHg/s) | 8919 ± 1209 | | 7197 ± 529 | 0.15 |
| dP/dt_min_ (mmHg/s) | -7146 ± 1435 | | -5578 ± 595 | 0.26 |
| Tau (ms) | 10.2 ± 1.9 | | 10.5 ± 1.4 | 0.89 |
|  |  | |  |  |
| *+ dobutamine* |  | |  |  |
| Heart rate (BPM) | 530 ± 31 | | 527 ± 16 | 0.93 |
| dP/dtmax (mmHg/s) | 11551 ± 1391 | | 9972 ± 900 | 0.33 |
| dP/dtmin (mmHg/s) | -8385 ± 1023 | | -6983 ± 722 | 0.26 |
| Tau (ms) | 7.1 ± 1.1 | | 8.4 ± 1.1 | 0.43 |
|  |  | |  |  |
| *Cine MRI* | | (n=6) | (n=6) |  |
| Age (weeks) | | 14.1 ± 0.3 | 14.8 ± 0.2 | 0.13 |
| Body weight (g) | | 23.5 ± 1.6 | 20.9 ± 0.6 | 0.16 |
| LV mass (mg) | | 79 ± 2 | 77 ± 3 | 0.53 |
| Heart rate (bpm) | | 387 ± 12 | 437 ± 20 | 0.06 |
| End-diastolic volume (µL) | | 51 ± 3 | 50 ± 3 | 0.62 |
| End-systolic volume (µL) | | 25 ± 2 | 25 ± 3 | 0.94 |
| Ejection fraction (%) | | 51 ± 4 | 51 ± 5 | 1.00 |
| Stroke volume (µL) | | 27 ± 2 | 25 ± 2 | 0.64 |
| Cardiac output (mL/min) | | 10.3 ± 1.1 | 10.8 ± 0.8 | 0.72 |

**Supplementary** **Table 6.** Left ventricular haemodynamics and MRI parameters in female mice prior to backcrossing. Data are mean ± SEM with inter-group comparison by Student’s t-test.

## Supplementary Figures


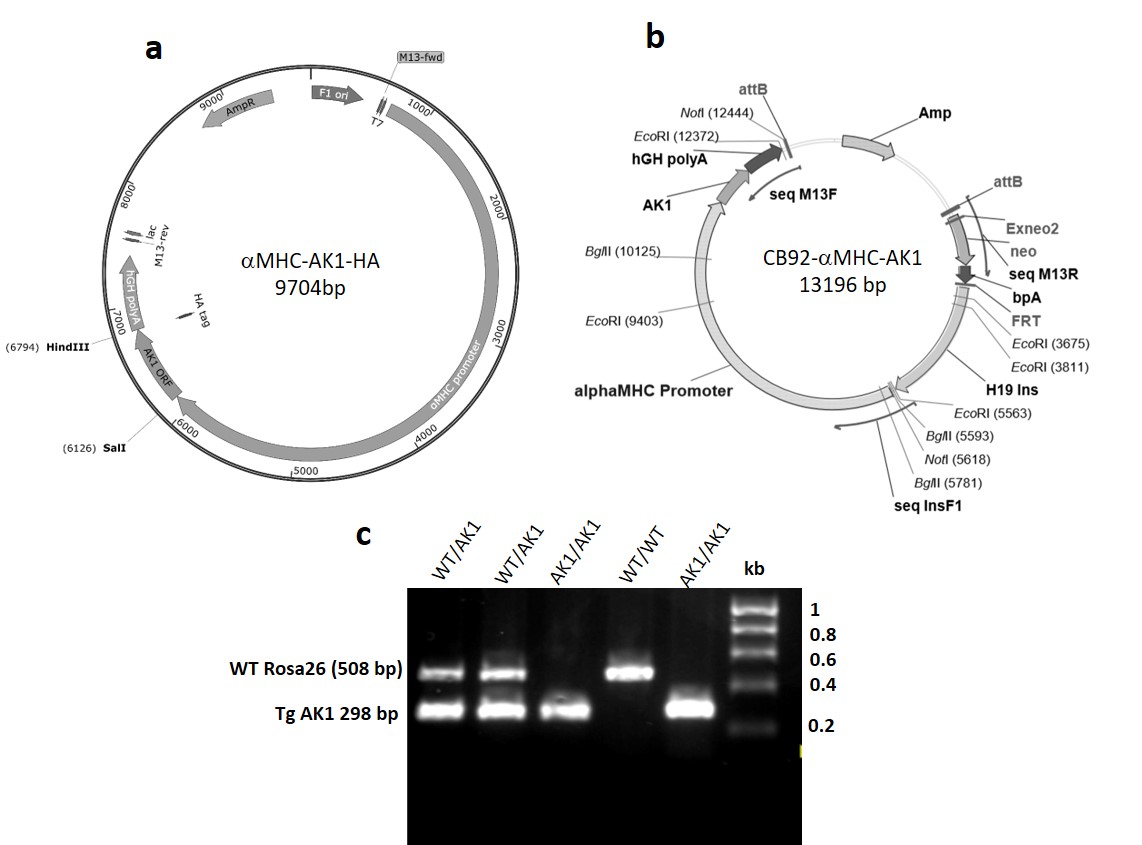


C

B

A

**Supplementary Figure 1. Generation of AK1-OE mice by Rosa26 targeted integration.** αMHC-AK1–HA construct **(A)**, with key features and restriction sites used for ligation (at *SalI*/*HindIII* cloning sites)*.* The 6763 bp fragment containing the αMHC promoter (5437bp), AK1 coding sequence (664bp), HA tag (27bp) and polyadenylation signal (635bp) was excised using *NotI* for cloning into the delivery vector shown in **(B)**; CB92–αMHC–AK1 delivery vector, with recombination sites for PhiC31–mediated integration (attB), neomycin resistance cassette for selection of stably transfected stem cells (neo), and insulator (H19 Ins). Note the *NotI* sites flanking the integrated construct. The delivery vector was used towards ROSA26 recombination using PhiC31 integrase as described before (2). AK1 transgene zygosity PCR **(C)**. The ROSA26 primers amplify a 508bp band in mice carrying the wild-type ROSA26 allele (i.e., WT/WT and WT/AK1 mice), while the AK1 primers amplify a 298bp band in mice carrying the transgene (i.e., WT/AK1 and AK1/AK1 mice). DNA Ladder (L) shows band size in kb. Panel (B) is courtesy of Dr. Ben Davies, WCHG, Oxford. Vector graphics were made using SnapGene^®^ Viewer 5.0.7.

L

E

F

HG

IG

JG

KG

GG

D

C

B

A

**Supplementary Figure 2.** Molecular verification of AK1 overexpression in tissue. Expression levels of AK1 transgene was confirmed in mouse LV from n=8 each wild-type, WT/WT; WT/AK1: heterozygote; AK1/AK1 homozygote for the AK1 transgene (***P*=0.0014 for WT/WT vs WT/AK1 and ****P*<0.0001 for WT/WT vs AK1/AK1) **(A)**. There was a gene-dosing effect seen as an increase WT/AK1 vs AK1/AK1 (**P*=0.03). As a result, total AK1 transcript was also increased in AK1-OE vs WT (**P*=0.0179 for WT/AK1 and ****P*<0.0001 for AK1/AK1) **(B)** whereas endogenous mRNA remained unchanged (One-way ANOVA *P*=0.9) **(C).** Data analysed by One-way ANOVA corrected for multiple comparisons. Expression of total AK1 transcript was examined across different tissues in n=3 WT and n=3 heterozygote OE (1M/2F for both groups). Comparisons between WT and OE per tissue by Student’s t test and significance shown for LV and atria (*P*=0.004 and *P*=0.0007, respectively) whereas AK1 mRNA levels in other tissues, were unchanged **(D)**. Transgenic AK1 protein was detected by immunoblotting in LV samples of WT/WT (n=8), WT/AK1 (n=7) and AK1/AK1 (n=4). Representative immunoblots for AK1 protein and beta tubulin, the latter was used as a loading control **(E).** Quantification of AK1 protein levels and analysis by one-way ANOVA *P*=0.1 **(F)**. AK2 protein expression detected at 26kDa in LV from WT and AK1-OE (OE; AK1/AK1) (**G**). The data are on n=5 each WT (2M/3F) and OE (3M/2F). Corresponding quantitation for AK2 protein normalized to β-tubulin **(H)**. AK3 protein levels in the same cohort as for AK2 detected at 29kDa **(I)** and quantitation of AK3/β-tubulin **(J).** Transcriptional levels were also examined by qRT-PCR, for the same n=5 cohort of each WT and OE for AK2 **(K)** and AK3 **(L)**. Mean values are shown ± SEM, Student’s t test for comparisons. *ns*: non-significant difference.

**REFERENCES**

1. Ng WA, Grupp IL, Subramaniam A, Robbins J. Cardiac myosin heavy chain mRNA expression and myocardial function in the mouse heart. *Circ Res* (1991) 68(6):1742-50. Epub 1991/06/01. PubMed PMID: 2036722.

2. Whittington HJ, Ostrowski PJ, McAndrew DJ, Cao F, Shaw A, Eykyn TR, et al. Over-expression of mitochondrial creatine kinase in the murine heart improves functional recovery and protects against injury following ischaemia-reperfusion. *Cardiovasc Res* (2018) 114(6):858-69. Epub 2018/03/07. doi: 10.1093/cvr/cvy054. PubMed PMID: 29509881; PubMed Central PMCID: PMCPMC5909653.

3. Livak KJ, Schmittgen TD. Analysis of relative gene expression data using real-time quantitative PCR and the 2(-Delta Delta C(T)) Method. *Methods* (2001) 25(4):402-8. Epub 2002/02/16. doi: 10.1006/meth.2001.1262. PubMed PMID: 11846609.

4. Gaussin V, Tomlinson JE, Depre C, Engelhardt S, Antos CL, Takagi G, et al. Common genomic response in different mouse models of beta-adrenergic-induced cardiomyopathy. *Circulation* (2003) 108(23):2926-33. Epub 2003/11/19. doi: 10.1161/01.CIR.0000101922.18151.7B. PubMed PMID: 14623810.

5. Wang X, Seed B. A PCR primer bank for quantitative gene expression analysis. *Nucleic Acids Res* (2003) 31(24):e154. Epub 2003/12/05. doi: 10.1093/nar/gng154. PubMed PMID: 14654707; PubMed Central PMCID: PMCPMC291882.

6. Faller KME, Atzler D, McAndrew DJ, Zervou S, Whittington HJ, Simon JN, et al. Impaired cardiac contractile function in arginine:glycine amidinotransferase knockout mice devoid of creatine is rescued by homoarginine but not creatine. *Cardiovasc Res* (2018) 114(3):417-30. Epub 2017/12/14. doi: 10.1093/cvr/cvx242. PubMed PMID: 29236952; PubMed Central PMCID: PMCPMC5982714.

7. Lygate CA, Bohl S, ten Hove M, Faller KM, Ostrowski PJ, Zervou S, et al. Moderate elevation of intracellular creatine by targeting the creatine transporter protects mice from acute myocardial infarction. *Cardiovasc Res* (2012) 96(3):466-75. Epub 2012/08/24. doi: 10.1093/cvr/cvs272. PubMed PMID: 22915766; PubMed Central PMCID: PMCPMC3500046.

8. Jiang CY, Yang KM, Yang L, Miao ZX, Wang YH, Zhu HB. A (1)H NMR-Based Metabonomic Investigation of Time-Related Metabolic Trajectories of the Plasma, Urine and Liver Extracts of Hyperlipidemic Hamsters. *PLoS One* (2013) 8(6):e66786. Epub 2013/07/11. doi: 10.1371/journal.pone.0066786. PubMed PMID: 23840531; PubMed Central PMCID: PMCPMC3694122.

9. Mayr M, Yusuf S, Weir G, Chung YL, Mayr U, Yin X, et al. Combined metabolomic and proteomic analysis of human atrial fibrillation. *J Am Coll Cardiol* (2008) 51(5):585-94. Epub 2008/02/02. doi: 10.1016/j.jacc.2007.09.055. PubMed PMID: 18237690.
